# Supplementary material for: Identification of the Ricin-B-Lectin LdRBLk in the Colorado Potato Beetle and an Analysis of Its Expression in Response to Fungal Infections
Source: J Fungi (Basel). 2021 May 6;7(5):364. doi: 10.3390/jof7050364 (PMC8148562; doi:10.3390/jof7050364)

Supplementary Materials

Identification of the Ricin-B-lectin LdRBLk in the Colorado potato beetle and an analysis of its expression in response to fungal infections

Table S1. Amino acid and nucleotide sequences of LdRBLk.

Title	Length	Sequence
ORF sequence	127aa	**M**SGIRIISKLSGLALDGNGSNVKAAMPNNTVGQIWSLESL GDGTFVIHSYSDKNMVLDIEYGCHGENIILYPRHGDDNQQ FYINSDGTIVSAGDRQKVLQMVRGKVVVRPVNISRNVDHI FNIEQLF
DNA sequence	381bp	ATGTCAGGTATTCGGATCATATCGAAATTATCTGGATTAG CTTTGGATGGAAATGGGAGCAACGTCAAAGCAGCAATGC CTAATAACACCGTTGGCCAAATTTGGTCTCTGGAAAGTTT GGGAGATGGAACTTTTGTCATACACAGTTATTCAGACAAG AACATGGTACTAGATATTGAATATGGCTGCCATGGCGAAA ATATCATACTATATCCAAGACATGGTGACGACAATCAACA GTTCTACATCAATTCTGACGGGACAATTGTATCAGCTGGTG ACAGACAGAAGGTTTTGCAAATGGTTAGAGGAAAAGTCGT GGTGCGACCAGTGAACATTTCGAGGAATGTCGATCACATTT TCAACATCGAGCAACTATTT
qPCR product sequence	134bp	TGGAAAGTTTGGGAGATGGAACTTTTGTCATACACAGTTATT CAGACAAGAACATGGTCCTAGATATTGAATATGGCTGCCAT GGTGAAAATATCATACTATATCCAAGACATGGTGACGACAA TCAACAGTTC

Table 2. List and description of genes and primers sequences used in the qPCR.

Gene Name	NCBI GenBank Accession number /Conserved Protein Domain Family/	Gene symbol	Primer sequence (5' – 3')	Product size (bp)	PCR efficiency (±SD)	Tm in qPCR, ºC	Primer's source
60S ribosomal protein L4	XM_023165859.1	*Rp4*	For GAAACGAGCATTGCCCTTCC<br>Rev TCGCTGACACTGTAGGGTTG	119	1,97±0,06	62	Shi et al (2013)*
60S ribosomal protein L18	XM_023172940.1	*Rp18*	For TAGAATCCTCAAAGCAGGTGGC<br>Rev CTGGACCAAAGTGTT CACTGC	133	1,96±0,04	62	Shi et al (2013)*
ADP-ribosylation factor-like protein 1	XM_023169879.1	*Arf19*	For CGGTGCTGGTAAAACGACAATATT<br>Rev TGACCTCCCAAATCCCAAACTT	135	1,95±0,06	62	Shi et al (2013)*
ADP-ribosylation factor 4	KC190027.1	*Arf2*	For GTGCTCGCGAACCATGTGA<br>Rev AAACCTCCAATCCCTCGTGAAG	140	2,00±0,01	62	Shi et al (2013)*
Ricin-B-lectin k	GEEF01084863.1 /pfam00652/	*LdRBlk*	For GAACTGTTGATTGTCGTCACCATG<br>Rev TGGAAAGTTTGGGAGATGGAACT	140	1,96±0,04	62	[a] ISaEA
Attacin_C domain contained protein	XM_023168834.1 /pfam03769/	*attacin*	For TGAGAACTCCACAAAATATTCACTTCG<br>Rev CTAAGGGTATGGCAGCAACAAC	98	1,90±0,07	62	[a] ISaEA
Heat shock protein 90	KC556802.1 /pfam00183 /	*hsp90*	For GGGTGTAGTCGACTCTGAAGAC<br>Rev AGAGCTCCTCAAACAGTTCCAA	125	1,90±0,06	62	[a] ISaEA

Shi, X.Q., Guo, W.C., Wan, P.J., Zhou, L.T., Ren, X.L., Ahmat, T., Fu, K.Y., Li, G.Q. Validation of reference genes for expression analysis by quantitative real-time PCR in *Leptinotarsa decemlineata* (Say). *BMC Res Notes* **2013**, *6*, 93 doi: 10.1186/1756-0500-6-93 * Primers were modified for this work by Rotskaya U. [a] Primers were designed by Rotskaya U. in programs Primer-BLAST https://www.ncbi.nlm.nih.gov/tools/primer-blast/, IDT OligoAnalyser Tool and IDT UNA Fold Tool https://eu.idtdna.com .

Figure S1. Tertiary structure of LdRBLk peptide constructed by I-TASSER program. The Model 1 (A) and the Model 2 (B).

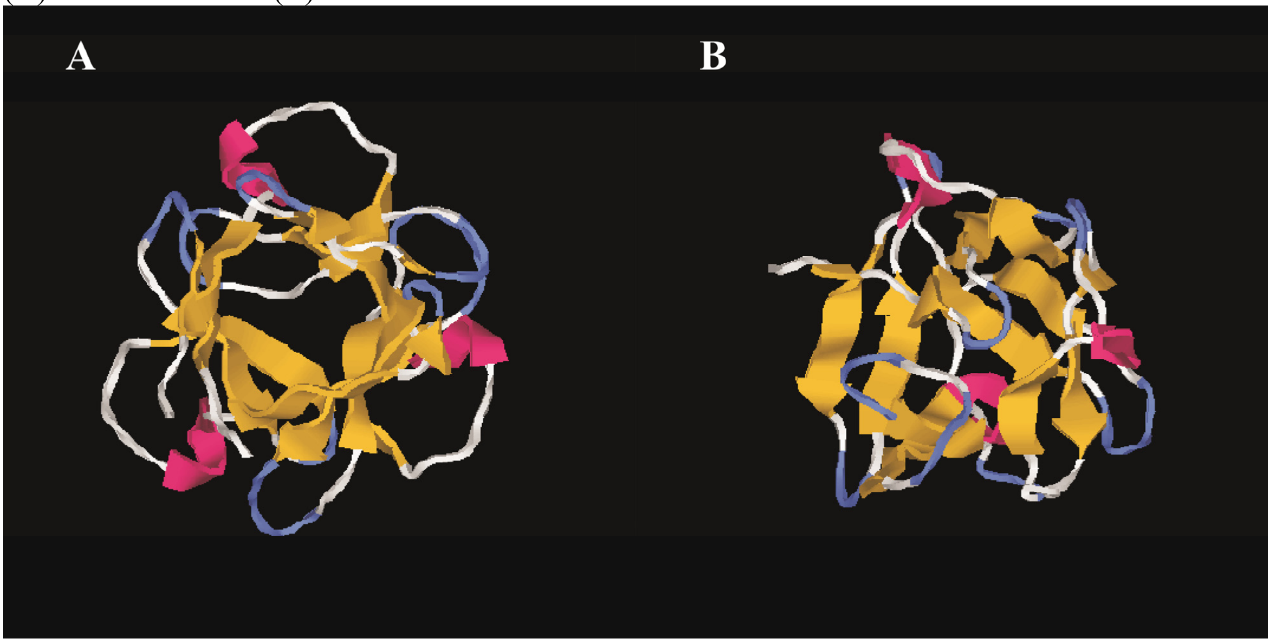

Figure S2. Analysis of the possibility of belonging of LdRBLk peptide to signal peptides determined by the online program SignalP-5.0 Server.

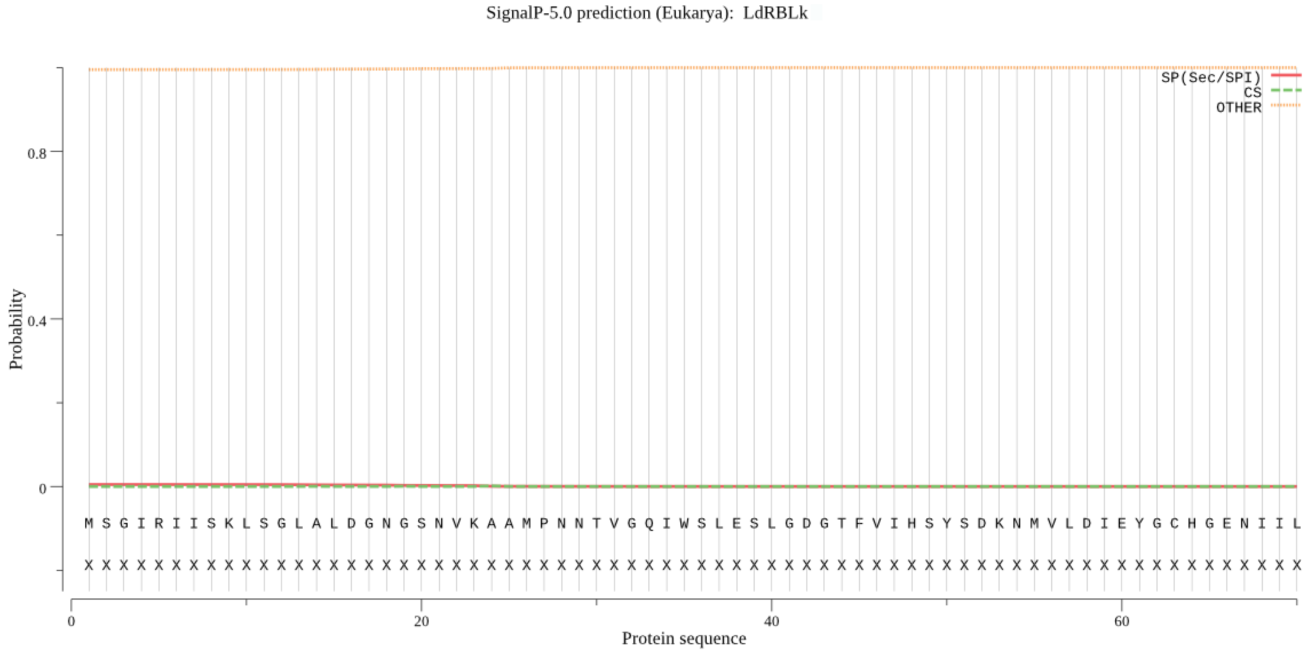

Figure S3. Prediction of the presence of a transmembrane domain in the LdRBLk peptide in the TMHMM online program. Possible transmembrane helices: inside to outside helices (i->o) 0 found, outside to inside helices (o->i) 0 found. Suggested models for transmembrane topology: no possible model found.

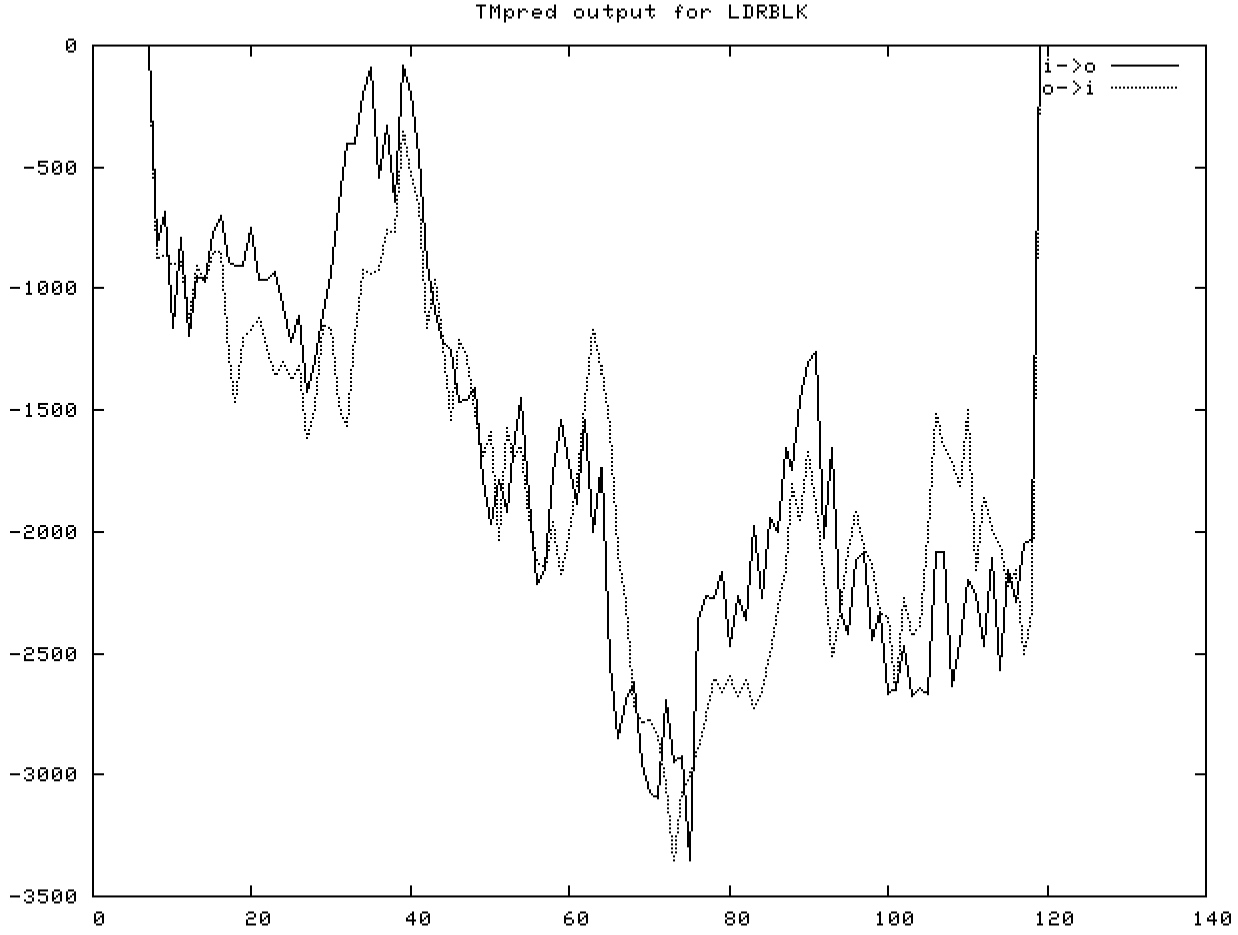

Figure S4. The analyze of hydrophilic property of LdRBLk peptide by the online program ProtScale using the Kyte-Doolittle (A) and the Hopp-Woods (B) algorithms.

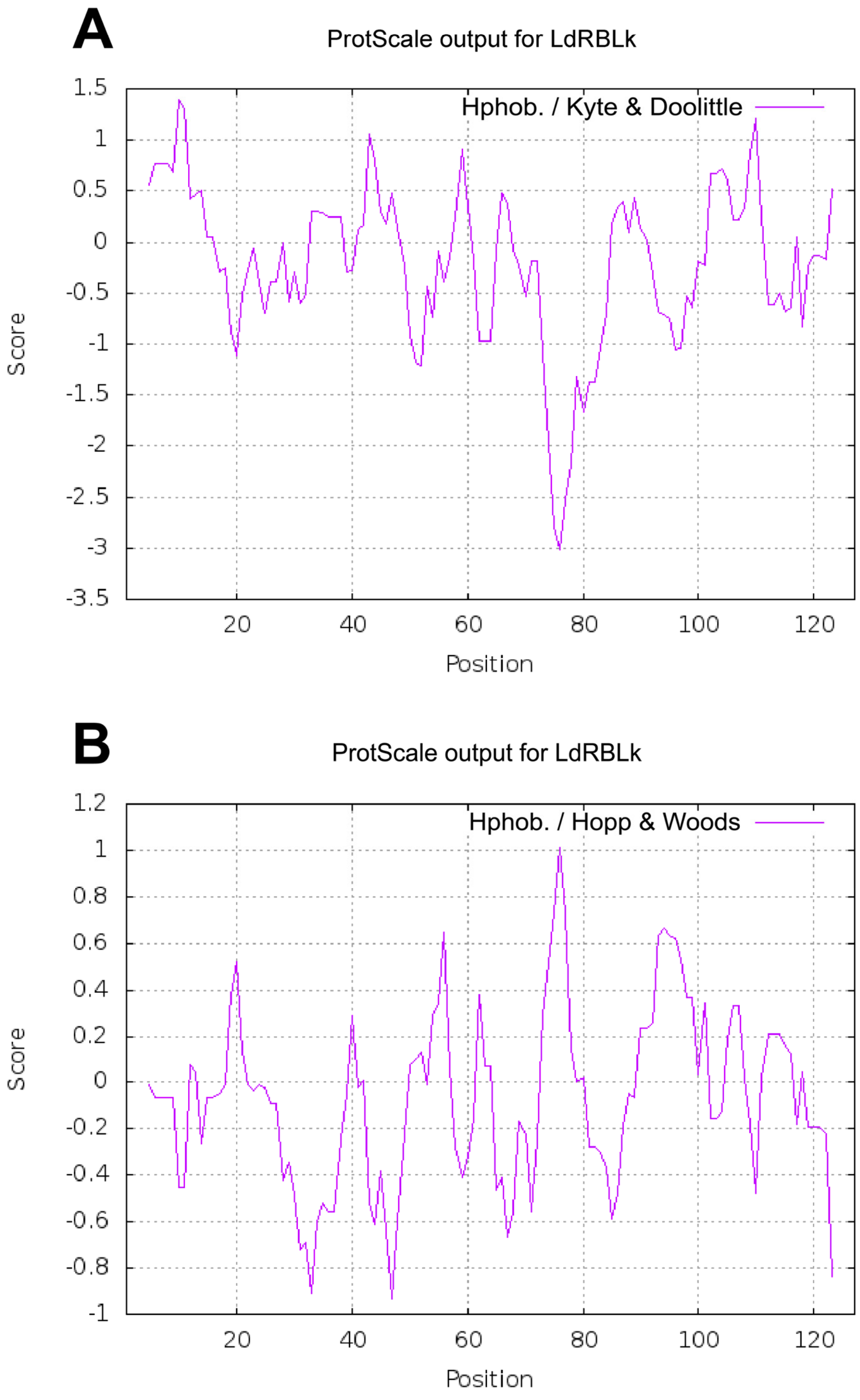

Figure S5. Multiple alignment of amino acid sequences of LdRBLs peptides of *L. decemlineata*. It was performed using the CLUSTAL algorithm in the MAFFT online program (v7.471).

```
CLUSTAL format alignment by MAFFT FFT-NS-i (v7.471)


LdRBLk          LVPS-----TFNIKMSG--------IRIISKLSGLALDGNGSNVKAAMPNNTVGQIWSLE
XP_023029736.1  MATS-PVILFFIICISSMYCLADDYVIIQSKTSGMVLDGNHYYVRARLADGSIGQKWNLT
XP_023029739.1  MASN-SAILCFFICISSMYCLANAYIIIESKNSGLVLDGNEYNVRAMPADGSIGQKWTLT
XP_023029750.1  MASNFSSYLFFLVVIAILTCLTEAQVRIKSKKTGLVFQYNGTNVEVAEPNDSEGQKWILE
                :...      * : ::         : * ** :*:.:: *   *..  .:.: ** * *

LdRBLk          SLGDGTFVIHSYSDKNMVLDIEYGCH-GE--NIILYPRHGDDNQQFYINSDGTIVSAGDR
XP_023029736.1  STKIGRFIITN-ARNGHVLDVQHGCKLGNTSNVIVYKNHFGRNQEFYINSDGTIVSSCDN
XP_023029739.1  PSDTGRFVITN-ARNGHALDVQDGCEKGN--NLIVYQKHSGTNQLFYINSDGTIGSACNT
XP_023029750.1  CSQKGHFIINS-NKQGLAMDIKKGCKIGQ--SVMVYDKHSRQNQQFFINSDATIGTACDA
                    * *:* .   :. .:*:: **. *:  .:::* .*   ** *:****.** :: :

LdRBLk          QKVLQMVRGKVVVRPVNIS-RNVDHIFNIEQLF
XP_023029736.1  NEIIEMKDGKVEVCGKEEPCSSGDVFFKILETK
XP_023029739.1  DLIVEMKDSNGQLGDIQNSCSSGDVIFEIQAAQ
XP_023029750.1  NLILLVKDRNVEVAPRDNY-FDFDIHFEIEPIE
                : :: :   :  :   :    . *  *:*
```

Figure S6. Multiple sequences alignment of Ricin-type β-trefoil lectin domain (pfam00652) contained proteins by hierarchical clustering algorithm.

Corpet, F. Multiple sequence alignment with hierarchical clustering. *Nucleic Acids Res*. **1988**, *16*, 10881-10890. doi:10.1093/nar/16.22.10881

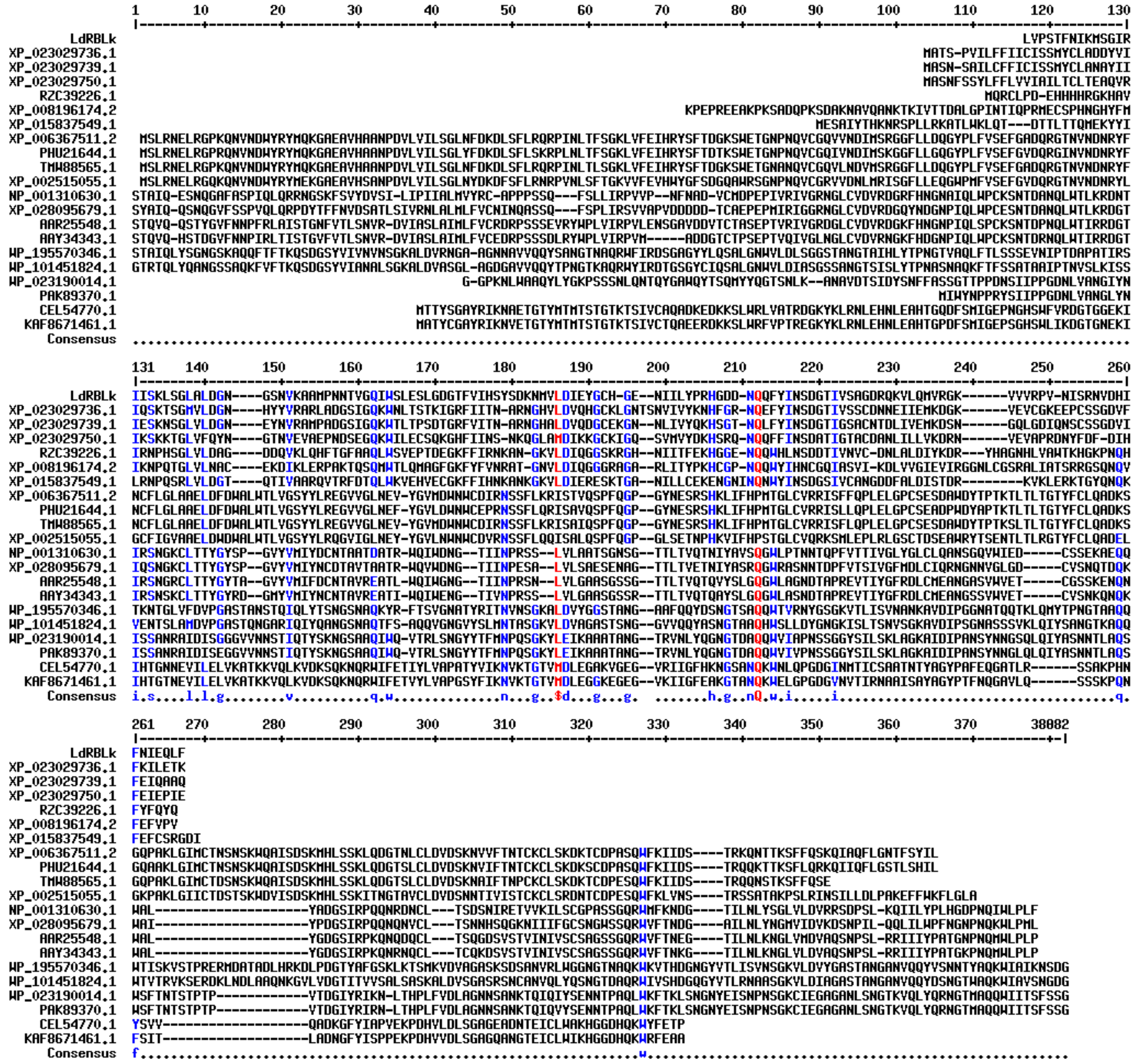

Figure S7. Predicted secondary and tertiary structure of β-lectin from *R. communis* ricin toxin (RBL). **A.** The secondary structure in the online SOPMA program. Strokes and lines are shown in blue, red, green and purple, representing an alpha helix, an extended strand, a beta turn, and a random coil, respectively. **B.** Tertiary structure of peptide constructed by I-TASSER program. The Model 1. **C.** Prediction of the ligand binding site for the RBL peptide by the I-TASSER program. The target ligand is galactose.

Sphyris, N., Lord, J.M., Wales, R., Roberts, L.M. Mutational analysis of the Ricinus lectin B-chains. Galactose-binding ability of the 2 gamma subdomain of Ricinus communis agglutinin B-chain. J Biol. Chem. **1995**, *35*, 20292-20297. doi: 10.1074/jbc.270.35.20292. PMID: 7657599.

A

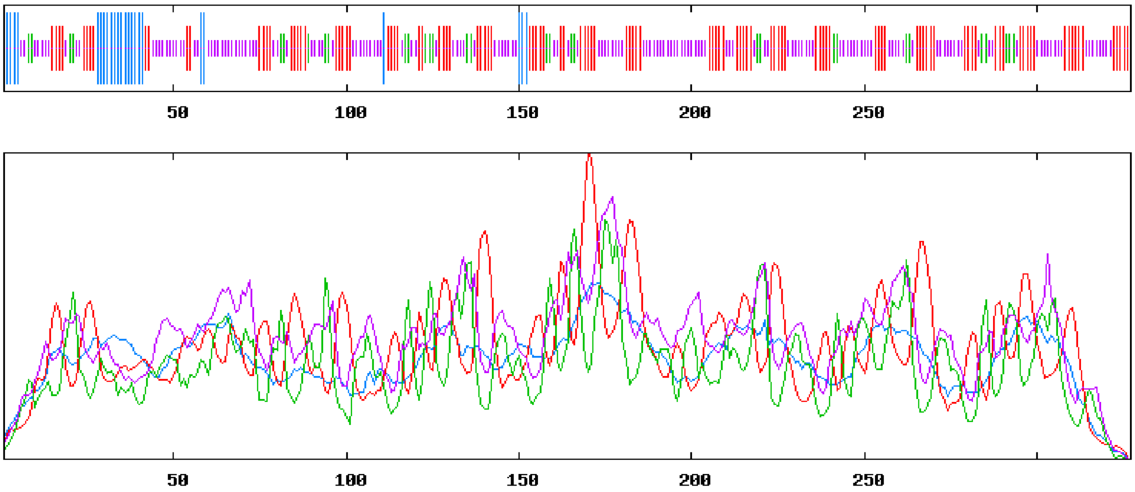

B **C**

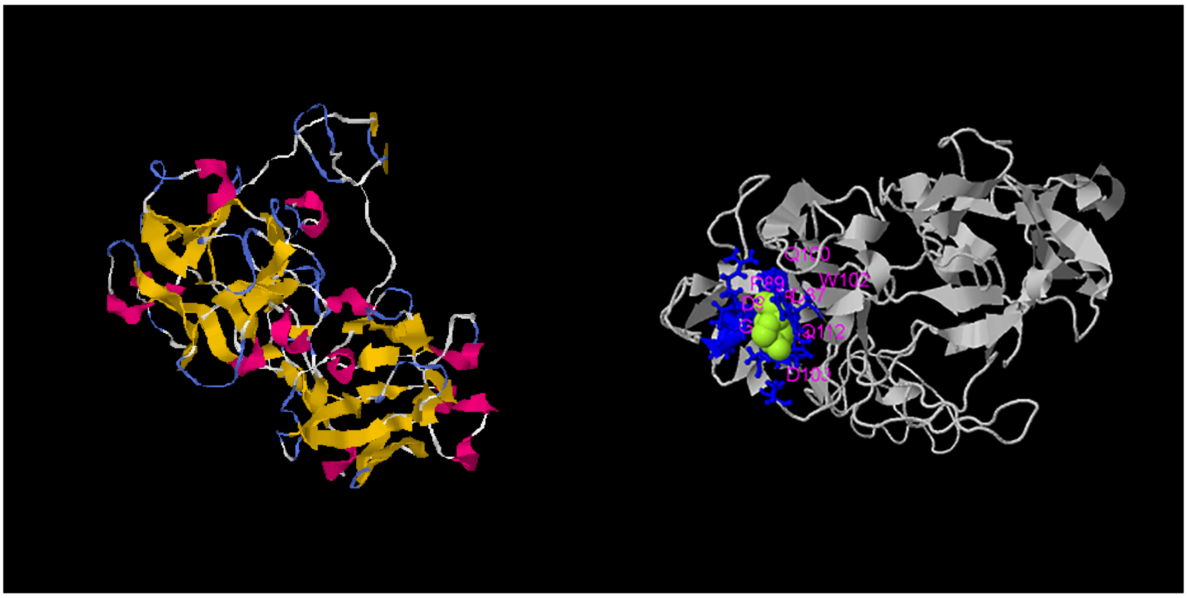

Supplement: Supplementary file 1 [file jof-07-00364-s001.zip › jof-1189084-supplementary.pdf]
